# Nickel Catalysts Supported on Acetylene Black for High-Efficient Electrochemical Oxidation and Sensitive Detection of Glucose

**DOI:** 10.1186/s11671-019-3218-1

**Published:** 2020-01-28

**Authors:** Xiaohui Gao, Wenshuai Feng, Yan Xu, Yifan Jiang, Cong Huang, Yougen Yi, Aimin Guo, Xiaoqing Qiu, Wei Chen

**Affiliations:** 1 School of Physics and Electronics, Hunan Key Laboratory for Super-Microstructure and Ultrafast Process, Central South University, Changsha, 410083 Hunan China; 2 College of Chemistry and Chemical Engineering, Central South University, Changsha, 410083 Hunan China; 3 State Key Laboratory of Electroanalytical Chemistry, Changchun Institute of Applied Chemistry, Chinese Academy of Sciences, Changchun, 130022 China; 4 University of Science and Technology of China, Hefei, 230029 Anhui China

**Keywords:** Nickel catalyst, Electrochemistry, Glucose oxidation, Glucose detection

## Abstract

**Abstract:**

Electrocatalytic glucose oxidation is a very important reaction in glucose fuel cell and medical diagnosis, which is limited by sluggish reaction kinetics and low diffusion coefficient. Herein, a composite (donated as Ni_6_/AB) consisting of atomically precise nickel catalyst with defined crystal structure [Ni_6_(SC_12_H_25_)_12_] and acetylene black(AB) has been initiated as a novel and high-efficient non-noble metal catalyst for the electrochemical oxidation of glucose benefiting from its high exposure of active sites and increased electron/mass transport. The present Ni_6_/AB composites display the onset potential of +1.24 V and the maximum current density of 5 mA cm^−2^ at the potential of +1.47 V in the electrolyte of 0.1 M KOH with 5 mM glucose. This electrochemical performance is much superior to the alone nickel catalysts, acetylene black, and previous reported nanomaterials. Furthermore, the obtained Ni_6_/AB composites are also expected to find important application in the electrochemical detection of glucose due to its high electrochemical performance. The sensitivity and the detection of limit are determined to be 0.7709 mA cm^−2^ mM^−1^ and 1.9 μM, respectively. Our study demonstrates that atomically precise nickel catalysts on acetylene black could be potential promising materials for next-generation energy devices and electrochemical sensors.

**Graphical abstract:**

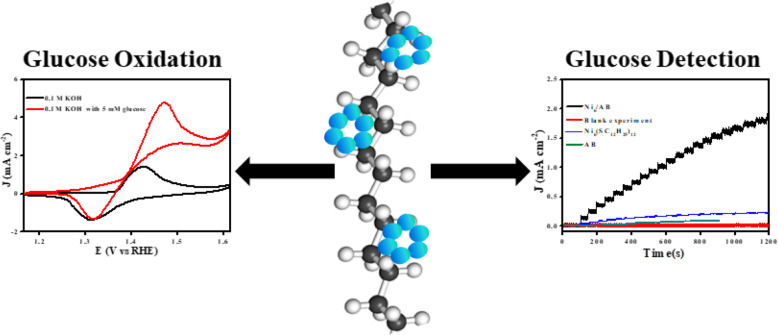

## Introduction

Recently, glucose fuel cell (GFC) has been considered as an ideal renewable energy device due to the non-toxicity, cleanliness, and huge theoretical energy density (4430 Wh/kg) [1–4]. However, the sluggish glucose oxidation kinetics and its low diffusion coefficient at the anode are the major obstacles that must be addressed for further development of the GFCs [5, 6]. On the other hand, the effective determination of glucose concentration is extremely necessary in the diagnosis and treatment of diseases such as diabetes and hyperglycemias, since glucose is vital living matter for human beings [7–9]. Currently, among the various analytical techniques for the detection of glucose, non-enzymatic electrochemical sensing methods are recognized as the most promising one because of the simple device design, high selectivity, and fast response ability [10–13]. Meanwhile, the sensitivity and detection limit of this method can be largely regulated and controlled by the electrode catalysts [14, 15]. Therefore, it is of great importance to develop high efficient electrocatalyst, especially with bifunctional properties for oxidation and detection of the glucose in the GFCs and glucose biosensors.

To date, different functional materials have been reported as electrocatalysts for the electrochemical oxidation and detection of glucose, including CuCo_2_S_4_ nanosheets grown on carbon fiber [16], platinum nanowires anchored on graphene-supported platinum nanoparticles [1], Co_3_O_4_ nanocrystals [17], and CuO nanorods derived from metal-organic framework. Recently, because the low valence state Ni(II) can be transformed to strong oxidizing high valence state Ni(III) species by electrochemically oxidation process, which can catalyze the glucose to gluconolactone and go back to Ni(II) at the same time, nickel-based materials gradually become a star catalyst for high efficient electrochemical glucose reaction [18–20]. For example, 1D NiMoO_4_ nanofibers designed by Wang et al were applied in the detection of glucose, exhibiting low oxidation potential (+ 0.5 V) and comparable sensitivity (193.8 μA mM^−1^ cm^−2^) [21]. To improve the glucose sensing performance, Xia et al prepared a three-dimensional flower-like Ni_7_S_6_ material with the sensitivity of 271.80 μA mM^−1^ cm^−2^ [22]. Furthermore, Xu et al increased the detection sensitivity to 346 μA mM^−1^ cm^−2^ on the basis of a novel Ni(OH)_2_@PEDOT-rGO nanocomposites as electrocatalysts for glucose sensing [23]. Nevertheless, to promote the practical application of GFCs and sensitive detection of glucose, the electrochemical performance of nickel-based electrocatalysts is still a large room for improvement.

Generally speaking, the electrochemical performances of the catalysts can be enhanced by increasing the number of catalytic active sites in material structural units [24]. This, in turn, means the preparation of electrocatalysts with abundant active sites, namely, metal nanoclusters, single-atom catalysts, and metalloploymer [25]. In recent years, owing to the high exposure of metal atoms and unambiguous geometric structure, atomically precise metal nanoclusters have attracted much attention from electrochemical glucose oxidation and sensing areas [26–31]. For example, He et al reported that the highly dispersed Pt nanoclusters anchored graphene composites showed the sensitivity of 71.9 μA mM^−1^ cm^−2^ and response potential of +0.1 V for glucose reaction [32]. Yang et al found that the alloy Pd-Au bimetallic nanoclusters can slightly increase the sensitivity to 75.3 μA mM^−1^ cm^−2^ [33]. Devoting to the efforts into non-noble metal nanoclusters, Chen et al studied that the sub-nanometer sized Cu_6_(GSH)_3_ clusters for electrochemical oxidation and detection of glucose, which exhibited the maximum oxidation peak of +0.5 V and the sensitivity of 15.1 μA mM^−1^ cm^−2^ [34]. Thus, it can be seen that the sensitivity of metal nanoclusters toward glucose sensing is still to be improved, especially that from non-noble metal nanoclusters. In addition, it is worthy to be mentioned that the stability of metal nanoclusters for electrochemical reaction should also be enhanced, which was not displayed in most of previous reports.

In this work, on the basis of the nickel-based materials’ inherent properties, high exposure of metal atoms in nanoclusters and extremely stable crown structure, an atomically precise nickel catalyst (Ni_6_(SC_12_H_25_)_12_) supported on acetylene black was examined for electrochemical glucose oxidation. The results showed that both of cyclic voltammetry and linear sweep voltammetry from Ni_6_/AB composites curves presented a prominent oxidation current peak in the electrolyte of 0.1 M KOH with 5 mM glucose in the potential range of +1.165 V to +1.615 V, when compared to the pure electrolyte of 0.1 M KOH. The onset potential and peak current position was respectively determined at about +1.24 V and around +1.47 V, which are much superior to that from the previous nickel-based materials. Moreover, based on the preeminent electrochemical activity, the electrode decorated by the present Ni_6_/AB composites also achieved the selectivity detection of glucose with the sensitivity of 0.7709 mA cm^−2^ mM^−1^, much higher than the value from most of the reports. We hope that the successful application of atomically precise nickel catalysts on acetylene black in glucose oxidation can push forward the development of GFCs and glucose sensors.

## Methods and experiments

### Chemicals and materials

Nickel nitrate hexahydrate (Ni(NO_3_)_2_^.^6H_2_O), dedecanethiol (C_12_H_25_SH), sodium borohydride (NaBH_4_), tetramethylene (THF), methanol (CH_3_OH), hexane (C_6_H_12_), dichloromethane (CH_2_Cl_2_, DCM), and ethanol (CH_3_CH_2_OH) were purchased from Aladdin. Tetra-n-octylammonium bromide ((C_8_H_17_)_4_NBr, TOABr) and acetylene black (AB) were bought from Alfa Aesar. Potassium hydroxide (KOH), glucose (C_6_H_12_O_6_), citric acid (C_6_H_8_O_7_, CA), uric acid (C_5_H_4_N_4_O_3_, UA), sucrose (C_12_H_22_O_11_, Suc), and sodium chloride (NaCl) were obtained from Beijing Chemical Reagent. The water used in the whole experiments was produced by a Water Purifier Ultrapure water system (18.25 MΩ cm). All glass vessels were immersed in aqua regia overnight and then rinsed with massive ultrapure water.

### Syntheses of the atomically precise nickel catalysts (Ni_6_(SC_12_H_25_)_12_)

Herein, the preparation of the atomically precise nickel catalysts, Ni_6_(SC_12_H_25_)_12_, follows the previous procedures used to synthesize Au_25_ and Ni_6_(SC_2_H_4_Ph)_12_ [35, 36]. Briefly, Ni(NO_3_)_2_^.^6H_2_O with the weight of 0.0623 g and 0.230 g of tetra-n-octylammonium bromide was co-dissolved in the 13 ml of THF. Then, 310 μL of dodecanethiol was added dropwise into the reaction system under the constant stirring. At this moment, the color of the mixture slowly changed from green to deep blue. After 3 h of constant stirring, 0.04 g of sodium borohydride in 5 ml ice water was poured to this solution and the reaction continued another 20 h. To obtain the product with high purity, THF was removed by rotary and evaporation, while methanol and DCM were used as washing and extracting solvent, respectively. The final product was dissolved in the DCM for further use.

### Syntheses of the Ni_6_(SC_12_H_25_)_12_ supported on acetylene black (donted as Ni_6_/AB composites)

Acetylene black is a frequently used support for metal nanocatalysts. In this work, to eliminate the possible metal impurity, acetylene black was firstly stirring in 1 M hydrochloric acid for 12 h. Then, a large amount of distilled water was used as washing solvent until the filtrate became neutral. The obtained acetylene black was dried in vaccum oven and waited for use in the next step.

For the preparation of Ni_6_/AB composites, 10 mg of the above handled acetylene black was dispersed in the 20 mL DCM. Then, 2 mL, 0.5 mg mL^−1^of Ni_6_(SC_12_H_25_)_12_ dichloromethane solution was introduced into the mixed system, and the reaction was allowed to proceed for 24 h under constant stirring. The final product of Ni_6_/AB composites was collected through the centrifugation and drying at room temperature.

### Characterizations

UV-Vis spectra were measured on a UV-3000PC spectrophotometer (Shanghai Mapada Instrumental Co., Ltd). X-Ray photoelectron spectroscopy (XPS) experiments were performed on an AVG Thermo ESCALAB 250 spectrometer (VG scientific) operated at 120 W. Fourier-transformed infrared spectroscopy (FTIR) tests were conducted on a VERTTEX 70 FTIR (KBr wafer technique). Matrix-assisted laser-desorption ionization time of flight mass spectrometric (MALDI-TOF MS) studies was completed by using a Bruker autoflexIII smartbeam MALDI-TOF/TOF-MS (Germany) and trans-2-[3-(4-tert-Butylphenyl)-2-methyl-2-propenyliene]malononitrile (DCTB) was used as the assisted matrix.

### Electrochemical measurements

The whole electrochemical measurements in this work were accomplished by using a CHI 760E electrochemical workstation in a standard three-electrode system at room temperature. During the experiments, 0.1 M KOH was utilized as the supporting electrolyte, while an Ag/AgCl (in saturated KCl) and a graphite electrode were taken as the reference and counter electrode, respectively. For the electrochemical oxidation of glucose, rotating disk electrode (5 mm diameter) coated with the catalyst ink (30 μL, 2 mg/mL) was used as working electrode. For the glucose sensing, the working electrode was a glassy carbon electrode (3 mm diameter) loaded with 2 mg/mL, 10-μL catalyst ink. The catalyst ink was the homogeneous mixture of 2 mg Ni_6_/AB(or Ni_6_(SC_12_H_25_)_12_, or AB), 0.5 ml ethanol, 0.5 ml water, and 20 μL Nafion. Herein, it is worthy to be mentioned that all current densities were normalized to the geometrical area of electrode, and all measured electrode potentials versus Ag/AgCl were transformed to a reversible hydrogen electrode (RHE) scale according to the following equation:

$$ E\left(V\;\mathrm{vs}\ \mathrm{RHE}\right)=E\left(V\;\mathrm{vs}\ \mathrm{Ag}/\mathrm{AgCl}\right)+0.059\ \mathrm{pH}+0.197 $$

## Results and discussion

### Synthesis and characterization of Ni_6_(SC_12_H_25_)_12_ and Ni_6_/AB composites

**Fig. 1:**
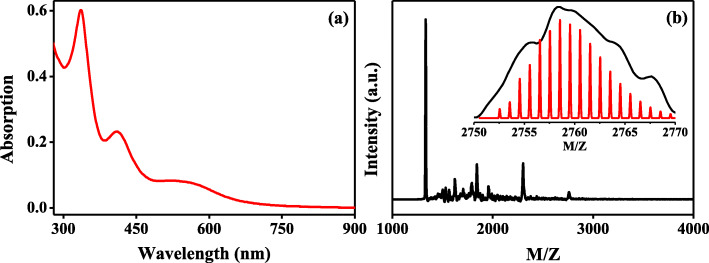
**a** UV-Vis absorption spectrum and **b** MALDI-TOF MS spectrum of the synthesized Ni_6_(SC_12_H_25_)_12_. The black curve and red curve in Fig. 1b inset represents experimental and simulated results, respectively

In the previous report, Ni_6_(SCH_2_CH_2_Ph)_12_ has been resolved to be a stable double-crown structure, and its UV-Vis spectrum clearly showed a discrete absorption curve [35]. Therefore, Ni_6_(SC_12_H_25_)_12_ is also expected to be a double-crown structure, which will provide high catalytic activity, high sensitivity and high electrochemical stability for the following glucose reaction. Herein, to demonstrate the successful synthesis of Ni_6_(SC_12_H_25_)_12_, UV-Vis absorption spectrum and objective spherical aberration correction field emission transmission electron microscope (FE-SEM) were employed to analyze the obtained product. As shown in Fig. 1a, resulted from the interband and intraband transitions, the UV-Vis absorption curve displays three obvious absorption bands located at 335 nm, 410 nm, and 540 nm. In the FE-TEM image shown in Additional file 1: Figure S1, the tiny particle circled in red can be obviously observed. These are similar with the previously reported results from Ni_6_(SC_2_H_4_Ph)_12_, suggesting the structural consistency between the two [35]. To obtain the precise composition of the product, matrix-assisted laser desorption ionization time-of-flight mass spectroscopy (MALDI-TOF MS) measurements were conducted and the results are presented in Fig. 1b. Obviously, the highest mass peak located at m/z 2759.06 was from the formula of [Ni_6_(SC_12_H_25_)_12_-10H]^+^. This indicates that the parent nickel nanocluster is Ni_6_(SC_12_H_25_)_12_, which is in the same form as the Ni_6_(SCH_2_CH_2_Ph)_12_. In MS spectrum, other smaller mass-charge ratio at the position of 2301.51, 1957.30, 1841.91, and 1623.31 can be ascribed to the fragments of parent catalyst, i.e, [Ni_5_(SC_12_H_25_)_10_-6H]^+^, [Ni_6_(SC_12_H_25_)_8_-5H]^+^, [Ni_4_(SC_12_H_25_)_8_-3H]^+^ and [Ni_4_(SC_12_H_25_)_7_-20H]^+^. Note that all the patterns from experimental mass spectrum could match well with that from the simulated one, although the accurate isotope distribution from test data was limited by instrument resolution, as shown in Fig. 1b inset and Additional file 1: Figure S2.

**Fig. 2:**
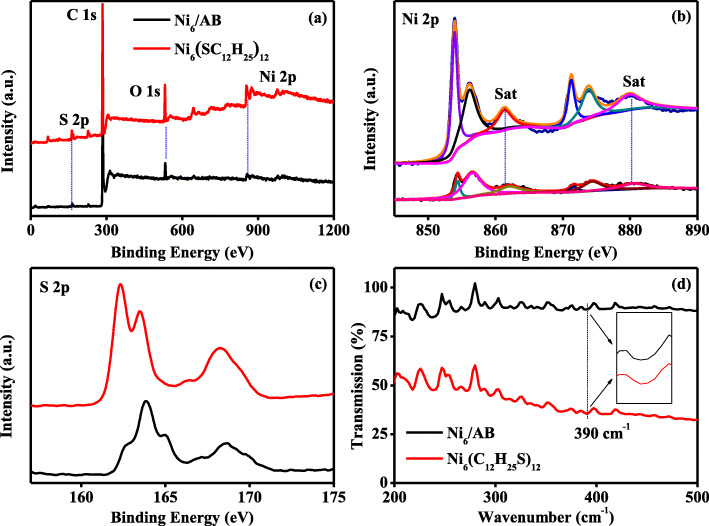
**a** XPS survey spectra of Ni_6_/AB composites (red line) and Ni_6_(SC_12_H_25_)_12_ (black line). **b** The Ni 2p deconvulted XPS spectrum of Ni_6_/AB composites (bottom one) and Ni_6_(SC_12_H_25_)_12_ (upper one). **c** The S 2p deconvulted XPS spectrum of Ni_6_/AB composites (bottom one, black line), and Ni_6_(SC_12_H_25_)_12_ (upper one, red line). **d** The FTIR spectra of Ni_6_/AB composites(red line) and Ni_6_(SC_12_H_25_)_12_ (black line). The inset in Fig. 2d is the amplifying region around 390 cm^−1^

For enhancing the subsequent electrochemical performance, the synthesized Ni_6_(SC_12_H_25_)_12_ in this work were supported on the acetylene black and were donated as Ni_6_/AB composites. Herein, X-ray photoelectronic energy spectroscopy (XPS) and far infrared spectroscopy (far IR) analysis were conducted to show the formation of Ni_6_/AB composites. As displayed in Fig. 2a, in agreement with the components of pure Ni_6_(SC_12_H_25_)_12_, the XPS survey spectra from Ni_6_/AB composites clearly provide the existence of Ni, S, C, and O element. As a comparison, XPS survey spectra of pure acetylene black only involves the elements of C and O, as shown in Additional file 1: Figures S3 and S4. This suggests the successful combination of Ni_6_(SC_12_H_25_)_12_ and acetylene black. To further analyze the possible interaction in Ni_6_/AB composites, the deconvoluted Ni 2p spectra from Ni_6_(SC_12_H_25_)_12_ and Ni_6_/AB composites were presented in Fig. 2b. The fitted components show that the binding energies at 853.9 and 856.1 eV can be assigned to the Ni 2p_3/2_, while the binding energies of 871.1 and 873.9 eV correspond to the Ni 2p_1/2_ [37, 38]. This indicates that the Ni(II) constituents are existing in Ni_6_(SC_12_H_25_)_12_ and Ni_6_/AB composites [39]. Interestingly, further observations find that the ratio of more positive Ni(II) component (the nickel with higher binding energy) to more negative Ni(II) component (the nickel with lower binding energy) apparently increases from 0.2:1 of Ni_6_(SC_12_H_25_)_12_ to 1:1 of Ni_6_/AB composites after loaded on the AB. More positive Ni(II) components may be favorable for subsequent catalytic reaction. Meanwhile, as shown in Fig. 2c, compared to that from Ni_6_(SC_12_H_25_)_12_, the S 2p signals from Ni_6_/AB composites also shift to the high energy direction. These imply that the electrons from metal nickel sites were shifting to AB supports, which will contribute to the enhancement of electrochemical performance, as discussed in following parts. In the far IR spectrum, as shown in Fig. 2d, the Ni-S absorption peak at 390 cm^−^1 simultaneously appears in the curves of Ni_6_/AB composites and Ni_6_(SC_12_H_25_)_12_, further evidencing the successful combination of nickel catalysts and support, in consistence with the result of XPS.

### Ni_6_/AB composites for electrochemical oxidation of glucose

**Fig. 3:**
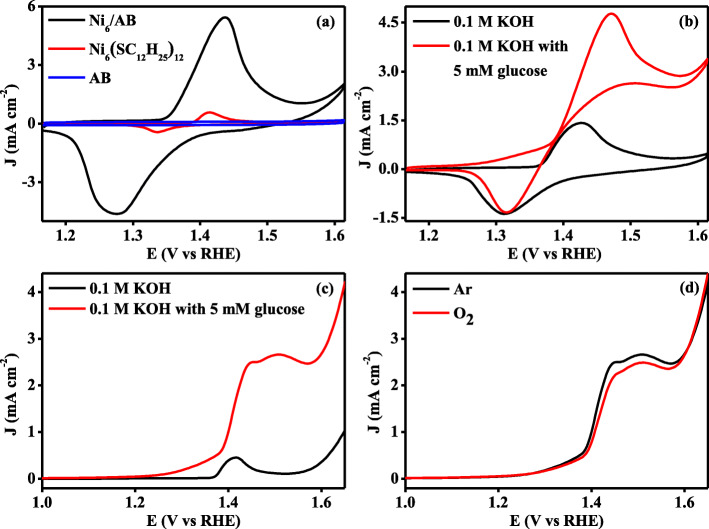
**a** Cyclic voltammtric curves of the Ni_6_/AB composites, Ni_6_(SC_12_H_25_)_12_, and AB in 0.1 KOH with the scan rate of 50 mV/s. **b** Cyclic voltametric curves of the Ni_6_/AB composites in 0.1 M KOH without or with the presence of 5 mM glucose with the scan rate of 50 mV/s. **c** Linear sweep voltametric curves of the Ni_6_/AB composites in 0.1 M KOH without or with the presence of 5 mM glucose with the scan rate of 10 mV/s. **d** Linear sweep voltametric curves of the Ni_6_/AB composites in 0.1 M KOH with the presence of 5 mM glucose under Ar or O_2_ atmosphere with the scan rate of 10 mV/s

As the result of unique properties, high exposed metal atoms and stable structure, the present Ni_6_/AB composites were investigated for the electrochemical oxidation of glucose, while pure Ni_6_(SC_12_H_25_)_12_ and acetylene black(AB) were used for comparison. In 0.1 M KOH, the typical cyclic voltammograms (CVs) of Ni_6_/AB composites, Ni_6_(SC_12_H_25_)_12_, and AB are presented in Fig. 3a. Compared to the featureless CV of AB, the CVs from Ni_6_/AB composites and Ni_6_(SC_12_H_25_)_12_ show a pair of distinct redox characteristic peaks in the range of scanning potential from + 1.2 V to + 1.6 V. Specifically, in the positive-potential scan, the current peak around + 1.44 V or + 1.41 V can be attributed to the oxidation of Ni(II) to Ni(III), and in the reverse scan, the reduction transformations of Ni(III) to Ni(II) can be respectively found at the potential of + 1.27 V and + 1.33 V for Ni_6_/AB composites and Ni_6_(SC_12_H_25_)_12_ [38, 40, 41]. Note that the existence of nickel signals in CV curve also illustrated the formation of Ni_6_/AB composites, in consistence with the results from XPS and far IR. For the oxidation of glucose, the CVs electrochemical results were presented in Fig. 3b. Apparently, when compared to CV curve in the pure electrolyte, the CV in 0.1 M KOH with 5 mM glucose evidently provides a more negative onset potential of + 1.26 V and a larger current density peak of 4.7 mA cm^−2^ at the potential of + 1.47 V for Ni_6_/AB composites. This shows the successful and efficient catalytic oxidation of glucose on the surface of the Ni_6_/AB composites-modified electrode, and the obtained results is much superior to that from the previous reported materials, as listed and compared in Additional file 1: Table S1. For the contrast materials of pure acetylene black, as shown in Additional file 1: Figure S5a, the current density difference from CV is very low in 0.1 M KOH with the presence of 5 mM glucose, when compared to that in 0.1 M KOH. For the alone Ni_6_(SC_12_H_25_)_12_, a clear glucose oxidation current peak and an onset oxidation potential at + 1.29 V(close to value of Ni_6_/AB composites) are shown in Additional file 1: Figure S5b. Combing the CV results from the three materials, it can be inferred that the Ni_6_/AB composites are high-efficient electrocatalysts for glucose oxidation, and the nickel sites are the main catalytic active sites. However, it is worthy to be noted that the peak current density of Ni_6_(SC_12_H_25_)_12_ is only around 1 mA cm^−2^ for the electrochemical oxidation of glucose, which is much lower than that of Ni_6_/AB composites.

To reveal the intrinsic activity, linear sweep voltammetry (LSV) was adopted to further examine the activity of Ni_6_/AB composites towards glucose electrochemical oxidation. As shown in Fig. 3c, in comparison with the curve in 0.1 M KOH, the LSV polarization curve from the Ni_6_/AB composites displays a prominent oxidation current in 0.1 M KOH with the presence of 5 mM glucose. Detailed analysis shows that the onset oxidation potential locates at + 1.24 V, in consistence with result from CV and much lower than that from other reported materials. Meanwhile, at the peak potential of + 1.47 V, the maximum current density is determined to be 2.67 mA cm^−2^ and increases 22 times compared to that from primary LSV in 0.1 M KOH, which is higher than that from the previous reports, for example, Nafion/GO_x_/PCP_1600_/GCE electrode and nanoporous Au [42, 43]. This once again demonstrates the Ni_6_/AB composites are highly active catalysts for electrochemical glucose oxidation. Moreover, as shown by the LSV and CV curves in Fig. 3d and Additional file 1: Figure S6, gas atmosphere does not have much influence on the onset potential and current density of glucose oxidation on the present catalysts, suggesting that the present Ni_6_/AB composites will be a kind of potential high-efficient electrocatalyst for the compartment-less fuel cell.

**Fig. 4:**
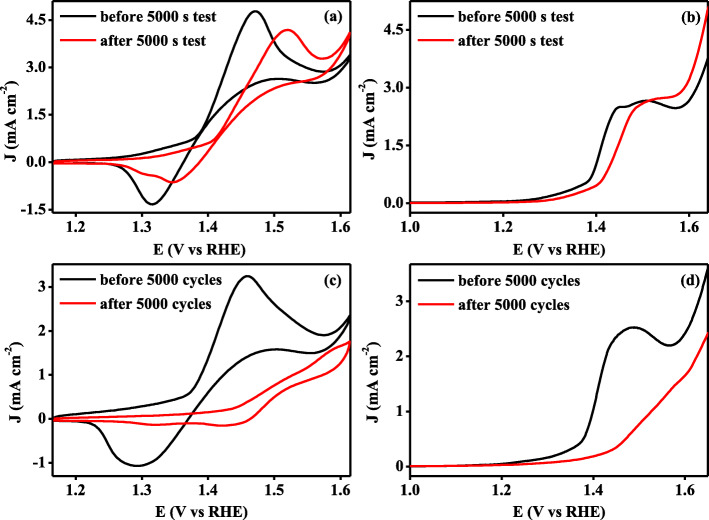
**a** Cyclic voltammetric curves and **b** linear sweep voltammetric curves of Ni_6_/AB composites before and after 5000 s *i*-*t* tests. **c** Cyclic voltammetric curves and **d** linear sweep voltammetric curves of Ni_6_/AB composites before and after 5000 cycles. The scan rate for CVs and LSVs is 50 mV/s and 10 mV/s, respectively

As we know, stability is another important factor for evaluating the electrochemical performance of catalysts. In this work, chronoamperometry was first employed to investigate the durability of Ni_6_/AB composites. As shown in Additional file 1: Figure S7, during 5000-s test at the potential of + 1.46 V, the current density from *i*-*t* curve of Ni_6_/AB composites decreases slowly from initial 1.6 to 1.13 mA cm^−2^. This may be mainly caused by the decrease in concentration of glucose and can be explained by following CV and LSV tests. As shown in Fig. 4a, the onset potentials from the CV before and after 5000 s test almost unchanged, while the value of current density peak only declined from 4.78 to 4.19 mA cm^−2^, i.e, remained around 90%. Similarly, the LSV curves before and after *i*-*t* test also have not changed much in terms of onset potential and maximum current density. As shown in Fig. 4b, both curves exhibited onset potential of + 1.26 V and the maximum current density of 2.64 mA cm^−2^ at the same potential. Such CV and LSV results give illustrations that the present Ni_6_/AB composites can allow 4 the durability of at least 5000 s. In addition, the stability examination through cyclic voltammetry was also conducted. As shown in Fig. 4c, after 5000 cycles in the potential window from + 1.165 to 1.615 V, the electrochemical signals from nickel redox electric pair and glucose oxidation approximately disappeared due to the dissolution of catalysts or the loss of Ni(III). Meanwhile, as shown in Fig. 4d, the same is true of the situation in LSV curves after 5000 cycles, signifying that the durability of Ni_6_/AB composites cannot exceed 5000 cycles. Therefore, the present Ni_6_/AB composites have a good stability for the electrochemical oxidation of glucose and may be a potential choice for GFCs.

Herein, with regard of the good catalytic performance from Ni_6_/AB composites, we can speculate three possible reasons: (1) the high exposure of nickel metal atoms in Ni_6_(SC_12_H_25_)_12_. As above mentioned, the geometric structure of Ni_6_(SC_12_H_25_)_12_ is a_._double-crown shape, so the whole nickel sites are exposed and stereospecific blockade can be ignored. This not only increases the catalytic active sites, but also enhances the availability of the sites; (2) the electron transfer between Ni_6_(SC_12_H_25_)_12_ and AB. As provided by XPS results, the more of high valence Ni(II) species in Ni_6_/AB composites may more easily transform into Ni(III) species, contributing to the electrochemical glucose oxidation, and (3) enhancement of conductivity and stability from AB support. It is well known that AB can usually improve the materials’ conductivity and its large surface can increase mass transport [44]. Thus, the high activity from Ni_6_/AB composites not only benefits from the high exposure of nickel sites, but also is largely promoted by the AB supports.

### Ni_6_/AB composites for electrochemical detection of glucose

**Fig. 5:**
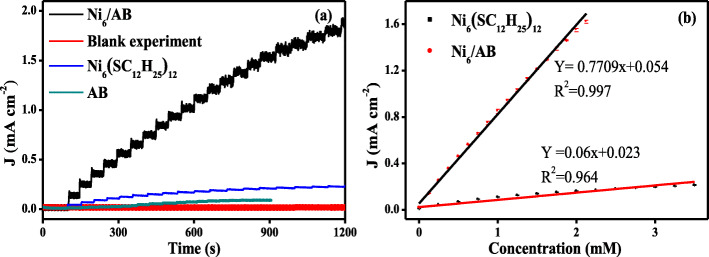
**a** The responsing current density-time curve (*J*-*t*) of the Ni_6_/AB composites, Ni_6_(SC_12_H_25_)_12_ and AB with the successive addition of glucose in 0.1 KOH at + 1.46 V. The *J*-*t* curve in 0.1 M KOH was used as blank control. **b** The linear relationship between the current density and concentration of glucose from *J*-*t* curves of Ni_6_/AB composites and Ni_6_(SC_12_H_25_)_12_

On the basis of their high performance towards the oxidation of glucose, the extra application of Ni_6_/AB composites in glucose sensing was further investigated through amperometry. During *i*-*t* curve tests, + 1.46 V was used as the working potential. As shown in Fig. 5a, relative to the control curve in 0.1 M KOH, the current density of experimental i-t curve increases step by step with the successive addition of 0.125 mM glucose into the electrolyte, which is much more evident than that from the alone Ni_6_(SC_12_H_25_)_12_ and AB. This suggests the rapid-response ability and high sensitivity of Ni_6_/AB composites towards glucose. As shown in Fig. 5b, the further data fitting between current density and the concentration of glucose exhibits a satisfying linear curve, and the relation equation can be expressed as: *J*(*y*) = 0.7709 × *C*(*x*) + 0.054 (*R*^2^ = 0.997), where *J* and *C* refers to the recording current density and the glucose concentration, respectively. From the relation equation, the sensitivity of Ni_6_/AB composites can be identified as 0.7709 mA cm^−2^ mM^−1^. Based on the signal-to-noise ratio of 3, the limit of detection of the present composite materials sensing system for glucose is calculated to be 1.9 μM. Thus, in aspect of the sensitivity and the detection limit, the present sensing system is much superior to that from the previous reports, as shown in Additional file 1: Table S2. As a comparison, the linear equation from pure Ni_6_(SC_12_H_25_)_12_ can be written as: *J*(*y*) = 0.06 × *C*(*x*) + 0.023 (*R*^2^ = 0.964), while the sensitivity and the limit of detection is determined to be 0.06 mA cm^−2^ mM^−1^ and 25 μM, respectively.

**Fig. 6:**
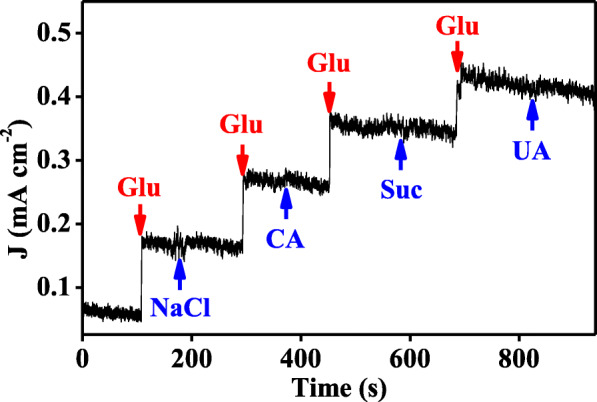
The current density responsing curves of the present Ni_6_/AB composites for the alternately addition of glucose (glu, 0.15 mM), NaCl (0.15 mM), CA (0.15 mM), Suc (0.025 mM), and UA (0.025 mM) in 0.1 M KOH

It is well known that sodium chloride (NaCl), citric acid (CA), sucrose (Suc), and uric acid (UA) usually disturb the detection of glucose in human blood serum. In this work, to investigate the selectivity of Ni_6_/AB composites, glucose (0.15 mM), NaCl (0.15 mM), CA (0.15 mM), Suc (0.025 mM), and UA (0.025 mM) were alternately introduced into the electrolyte of 0.1 M KOH. As shown in Fig. 6, other four analytes did not produce observable current signals under the same condition, and the succedent reponsing current of glucose was also not affected by these disturbing substances. Meanwhile, it is more worthy to be noted that the healthy physiological concentration of glucose is much higher than that from above mentioned interfences in human body [45]. Therefore, these results indicate the high selectivity of Ni_6_/AB composites towards glucose sensing and its potential practical application in glucose sensors.

## Conclusions

In this work, Ni_6_/AB composites consisting of atomically precise Ni_6_(SC_12_H_25_)_12_ and acetylene black were investigated as catalysts for the electrochemical oxidation of glucose. The results from cyclic voltammetric and linear sweep voltammetric curves demonstrated the high-efficient performance of Ni_6_/AB composites for glucose oxidation. Combining electrochemical data with XPS results, the high catalytic activity of present composites can be attributed to the high-exposed nickel active sites and increased mass/electron transportation. In addition, based on the high electrochemical activity, the obtained Ni_6_/AB composites can also realize the sensitivity and selectivity detection of glucose. We hope this work could not only lay a foundation for the application of atomically precise catalysts in GFCs and glucose sensors, but also promote the development of nickel-based catalysts in other fields such as disease diagnosis, microbiological fuel cell, and gas sensing.

## Supplementary information

**Additional file 1: Figure S1.** The objective spherical aberration correction field emission transmission electron microscopy from parent nickel catalyst, Ni_6_(SC_12_H_25_)_12_. The scale bar is 10 nm. **Figure S2.** The experimental and simulated patterns for the fragments from parent nickel catalyst, Ni_6_(SC_12_H_25_)_12_. **Figure S3.** The XPS survey spectrum of pure acetylene black. **Figure S4.** (a) The deconvulted C 1s XPS spectrum from AB; (b) The deconvulted O 1s XPS spectrum from AB; (c) The deconvulted S 2p XPS spectrum from AB; (a) The deconvulted Ni 2p XPS spectrum from AB. **Figure S5.** Cyclic voltametric curves of AB (left) and Ni_6_(SC_12_H_25_)_12_ (right) in 0.1 M KOH with the presence of 5 mM glucose with the scan rate of 50 mV/s. **Figure S6.** Cyclic voltametric curves of the Ni_6_/AB composites in 0.1 M KOH with the presence of 5 mM glucose under Ar or O_2_ atomsphere with the scan rate of 50 mV/s. **Figure S7.** The current density-time curve of the Ni_6_/AB composites in 0.1 M KOH with the presence of 5 mM glucose during 5000s i-t test. **Table S1.** Comparison of the Ni_6_/AB composites with the previously reported electrocatalysts for glucose oxidation. **Table S2.** Comparison of the Ni_6_/AB composites with the previously reported material for glucose sensing

## Data Availability

The data and conclusions in this work are all showed in this paper.

## Abbreviations

AB: Acetylene black
CA: Citric acid
CV: Cyclic voltammograms
DCM: Dichloromethane
GFC: Glucose fuel cell
LSV: Linear sweep voltammetry
Suc: Sucrose
THF: Tetremethylene
TOABr: Tetra-n-octylammonium bromide
UA: Uric acid
