# Supplementary material for: Nickel Catalysts Supported on Acetylene Black for High-Efficient Electrochemical Oxidation and Sensitive Detection of Glucose
Source: Nanoscale Res Lett. 2020 Jan 28;15:23. doi: 10.1186/s11671-019-3218-1 (PMC6987276; doi:10.1186/s11671-019-3218-1)
Supplement: Supplementary file 1 — Additional file 1: Figure S1. The objective spherical aberration correction field emission transmission electron microscopy from parent nickel catalyst, Ni6(SC12H25)12. The scale bar is 10 nm. Figure S2. The experimental and simulated patterns for the fragments from parent nickel catalyst, Ni6(SC12H25)12. Figure S3. The XPS survey spectrum of pure acetylene black. Figure S4. (a) The deconvulted C 1s XPS spectrum from AB; (b) The deconvulted O 1s XPS spectrum from AB; (c) The deconvulted S 2p XPS spectrum from AB; (a) The deconvulted Ni 2p XPS spectrum from AB. Figure S5. Cyclic voltametric curves of AB (left) and Ni6(SC12H25)12 (right) in 0.1 M KOH with the presence of 5 mM glucose with the scan rate of 50 mV/s. Figure S6. Cyclic voltametric curves of the Ni6/AB composites in 0.1 M KOH with the presence of 5 mM glucose under Ar or O2 atomsphere with the scan rate of 50 mV/s. Figure S7. The current density-time curve of the Ni6/AB composites in 0.1 M KOH with the presence of 5 mM glucose during 5000s i-t test. Table S1. Comparison of the Ni6/AB composites with the previously reported electrocatalysts for glucose oxidation. Table S2. Comparison of the Ni6/AB composites with the previously reported material for glucose sensing [file 11671_2019_3218_MOESM1_ESM.doc]

Electronic Supplementary Material

| **Nickel catalysts supported on acetylene black for high-efficient electrochemical oxidation and sensitive detection of glucose** |  |
| --- | --- |
| Xiaohui Gao^1,2^, Wenshuai Feng^1,2^, Yan Xu^2^, Yifan Jiang^2^, Cong Huang^2^, Yougen Yi^1^, Xiaoqing Qiu^2^(**🖂**), Wei Chen^3,4^(**🖂**)  *^1^ School of Physics and Electronics, Hunan Key Laboratory for Super-Microstructure and Ultrafast Process, Central South University, Changsha 410083, Hunan, China*  *^2^ College of Chemistry and Chemical Engineering, Central South University, Changsha 410083, Hunan, China*  *^3^ State Key Laboratory of Electroanalytical Chemistry, Changchun Institute of Applied Chemistry, Chinese Academy of Sciences, Changchun 130022, China*  *^4^ University of Science and Technology of China, Hefei 230029, Anhui, China*  *Supporting information to DOI 10.1007/s12274-****-****-** |  |


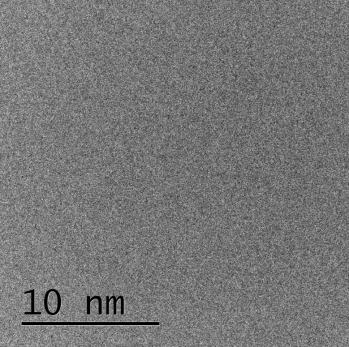


|  |
| --- |
| Address correspondence to Xiaoqing Qiu, xq-qiu@csu.edu.cn; Wei Chen, weichen@ciac.ac.cn |

**Figure S1** The objective spherical aberration correction field emission transmission electron microscopy from parent nickel catalyst, Ni_6_(SC_12_H_25_)_12_. The scale bar is 10 nm.

**Figure S2** The experimental and simulated patterns for the fragments from parent nickel catalyst, Ni_6_(SC_12_H_25_)_12_.

**Figure S3** The XPS survey spectrum of pure acetylene black.

**Figure S4** (a) The deconvulted C 1s XPS spectrum from AB; (b) The deconvulted O 1s XPS spectrum from AB; (c) The deconvulted S 2p XPS spectrum from AB; (a) The deconvulted Ni 2p XPS spectrum from AB;

**Figure S5** Cyclic voltametric curves of AB (left) and Ni_6_(SC_12_H_25_)_12_ (right) in 0.1 M KOH with the presence of 5 mM glucose with the scan rate of 50 mV/s.

**Figure S6** Cyclic voltametric curves of the Ni_6_/AB composites in 0.1 M KOH with the presence of 5 mM glucose under Ar or O_2_ atomsphere with the scan rate of 50 mV/s.

**Table S1** Comparsion of the Ni_6_/AB composites with the previously reported electrocatalysts for glucose oxidation.

Catalysts Electrolyte Glucose concentration Peak current density Ref.

ZMNPs^[a]^  0.1 M NaOH 10.0 mM about 6 μA Masoud[^25^](#_ENREF_43)

Ni_4_Co_2_ catalysts 3 M KOH 1.0 M 1.5 mA Zhang et al[^26^](#_ENREF_44)

Hollow CuCo_2_O_4_ 0.2 M KOH 1.2 mM 150 μA Hu et al[^27^](#_ENREF_45)

Fe_15_Pt_85_ NPs 0.1 M PBS^[b]^ 50 mM 6 mA cm^−2^ Soltanian et al [^28^](#_ENREF_46)

FAD-GDH/CNT McIlvaine [buffer](https://www.sciencedirect.com/topics/chemistry/buffer-solution)^[c]^  100 mM 4.7 mA cm^−2^  Holzinger et al[^29^](#_ENREF_47)

Ni_6_/AB composites 0.1 M KOH 5 mM 4.7 mA cm^-2^ this work

^[a]^ represents znic molybdate nanostrucutre, ^[b]^ pH = 7, ^[c]^ pH = 7.

**Figure S7** The current density-time curve of the Ni_6_/AB composites in 0.1 M KOH with the presence of 5 mM glucose during 5000s i-t test.

**Table S2** Comparion of the Ni_6_/AB composites with the previously reported material for glucose sensing.

Catalysts Electrolyte Sensitivity Detection limit(μM) Ref

CS-NiOOH/GC 0.1 N NaOH 687 μA mM^−1^ cm^−2^ 0.02 Roth et al[^30^](#_ENREF_48)

Co_3_O_4_@ Carbon 0.1 KOH 249.1 μA mM^−1^ cm^−2^ 5 Hu et al[^31^](#_ENREF_49)

AuNP/NG 0.1 M NaOH 0.25 μA mM^−1^ cm^−2^ 12  Lee et al[^32^](#_ENREF_50)

Cu NC/ITO 0.1 M KOH 151 μA mM^−1^ cm^−2^ 4.07 Chen et al[^17^](#_ENREF_31)

Graphene/Co_3_O_4_ Composites 0.1 M NaOH 122.1 µA mM^−1^ cm^−2^ 0.157 Xu et al[^33^](#_ENREF_51)

Ni_6_/AB composites 0.1 M KOH 770.9 µA mM^-1^ cm^-2^  1.9 this work
